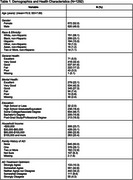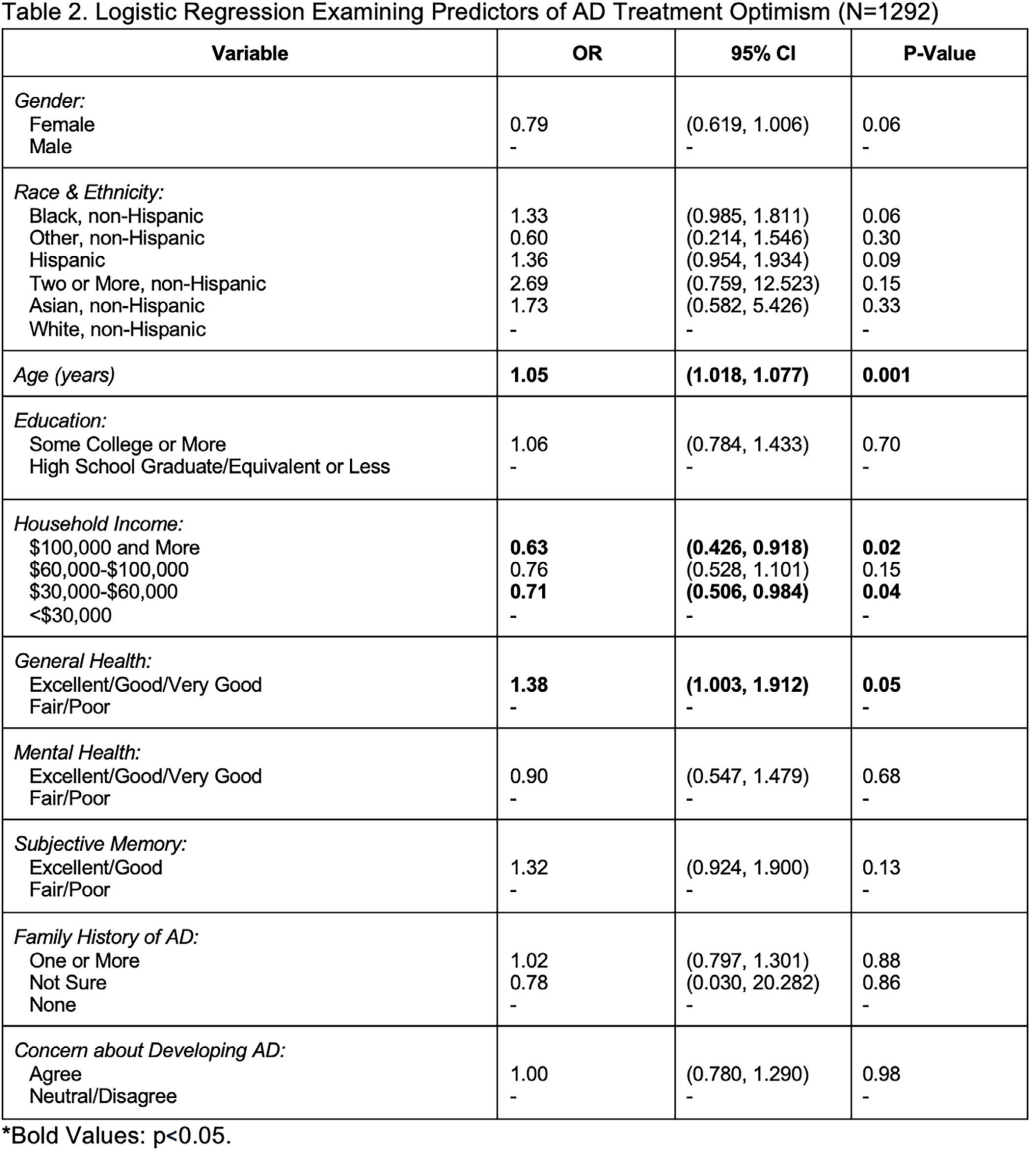# Predictors of Alzheimer's Disease Treatment Optimism Among a Nationally Representative Sample of Older Adults

**DOI:** 10.1002/alz70858_107068

**Published:** 2025-12-25

**Authors:** Sarah McCain, Jonathan M Reader, J. Scott Roberts

**Affiliations:** ^1^ University of Michigan School of Public Health, Ann Arbor, MI, USA; ^2^ Michigan Alzheimer's Disease Research Center, Ann Arbor, MI, USA

## Abstract

**Background:**

The health psychology literature suggests that treatment optimism (i.e., the belief that disease‐specific treatments’ efficacy and availability will significantly improve over time) may predict help‐seeking and influence medical decisions (e.g., disease screening and early detection). However, little is known about treatment optimism regarding Alzheimer's disease (AD). This secondary data analysis describes AD treatment optimism and its predictors among a nationally representative sample of older adults in the US.

**Method:**

The National Poll on Healthy Aging (NPHA), a recurring, nationally representative survey of adults aged 50 and older, conducted a cross‐sectional internet and telephone survey in 2023 using NORC's AmeriSpeak Panel®. Survey measures included self‐reported demographic/health characteristics (e.g., family history of AD) and dementia‐related perceptions (e.g., concern about developing AD, subjective memory rating). The main outcome measure assessed AD treatment optimism by asking respondents to rate their agreement (1=*Strongly Disagree* to 5=*Strongly Disagree*) with the statement: “AD will one day become a manageable chronic condition like diabetes or heart disease.” Logistic regression modeling identified predictors of treatment optimism, utilizing a significance value of *p* <0.05.

**Result:**

The sample included 1,292 respondents, with a mean age of 70.9 years (range: 50‐80; SD: 7.89) and a majority reporting as female (52.0%) and non‐Hispanic white (58.1%) (Table 1). Most (52.9%) respondents agreed that AD would one day become a manageable condition, with 10.3% strongly agreeing. AD treatment optimism was associated with older age (OR: 1.05; 95% CI: 1.018, 1.077), self‐reported good to excellent general health (OR: 1.38; 95% CI: 1.003, 1.912), and household income <$30,000 ($30,000‐$60,000: OR: 0.71, 95% CI: 0.506, 0.984; >$100,000: OR: 0.63, 95% CI: 0.426, 0.918) (Table 2). AD treatment optimism did not differ significantly by gender, race/ethnicity, education, mental health, family history of AD, concern about developing AD, or subjective memory rating.

**Conclusion:**

Findings from this nationally representative survey suggest high overall levels of treatment optimism among US older adults, with treatment optimism significantly differing by age, health status, and household income. Future research is needed to determine how older adults’ treatment optimism changes in the face of improved therapies and influences their engagement in emerging AD screening and therapeutic options.